# Identification of new biosignatures for clinical outcomes in stable coronary artery disease - The study protocol and initial observations of a prospective follow-up study in Taiwan

**DOI:** 10.1186/s12872-017-0471-z

**Published:** 2017-01-28

**Authors:** Hsin-Bang Leu, Wei-Hsian Yin, Wei-Kung Tseng, Yen-Wen Wu, Tsung-Hsien Lin, Hung-I Yeh, Kuan-Cheng Chang, Ji-Hung Wang, Chau-Chung Wu, Jaw-Wen Chen

**Affiliations:** 1 Institute of Clinical Medicine and Cardiovascular Research Center, National Yang-Ming University, Taipei, Taiwan; 2 Heath Care and Management Center, Taipei Veterans General Hospital, Taipei, Taiwan; 3 Divison of Cardiology, Department of Medicine, Taipei Veterans General Hospital, Taipei, Taiwan; 4 Division of Cardiology, Heart Center, Cheng-Hsin General Hospital, and School of Medicine, National Yang-Ming University, Taipei, Taiwan; 5 Department of Medical Imaging and Radiological Sciences, I-Shou University, Kaohsiung, Taiwan; 6 Division of Cardiology, Department of Internal Medicine, E-Da Hospital, Kaohsiung, Taiwan; 7 Cardiology Division of Cardiovascular Medical Center and Department of Nuclear Medicine, Far Eastern Memorial Hospital, New Taipei City, Taiwan; 8 Division of Cardiology, Department of Internal Medicine, Kaohsiung Medical University Hospital and Kaohsiung Medical University, Kaohsiung, Taiwan; 9 Mackay Memorial Hospital, Mackay Medical College, New Taipei City, Taiwan; 10 Division of Cardiology, Department of Internal Medicine, China Medical University Hospital, Taichung, Taiwan; 11 Graduate Institute of Clinical Medical Science, China Medical University, Taichung, Taiwan; 12 Department of Cardiology, Buddhist Tzu-Chi General Hospital, Tzu-Chi University, Hualien, Taiwan; 13 Division of Cardiology, Department of Internal Medicine, National Taiwan University College of Medicine and Hospital, Taipei, Taiwan; 14 Department of Primary Care Medicine, College of Medicine, National Taiwan University, Taipei, Taiwan; 15 Institute of Pharmacology, National Yang-Ming University School of Medicine, Taipei, Taiwan; 16 Division of Clinical Research, Department of Medical Research, Taipei Veterans General Hospital, 201, Section 2, Shih-Pai Road, Taipei, 112 Taiwan, R.O.C.

**Keywords:** Asian, Biomarkers, Biosignaturs, Chinese, Coronary artery disease, Lipid, Outcome

## Abstract

**Background:**

Either classic or novel biomarkers have not been well investigated for clinical outcomes of coronary artery disease (CAD) in Asian people especially ethnic Chinese. We reported here a prospective national-based follow-up study that aims to elucidate the clinical profiles and to identify the new biosignatures (especially the non-lipid profile and inflammatory biomakers) for future clinical outcomes in a sizable cohort of stable CAD patients in Taiwan.

**Methods:**

A total of 2500 CAD patients under stable condition after successful percutaneous coronary intervention will be enrolled for clinical data collection and blood/urine sampling in northern, southern, western, or eastern part of Taiwan between 2012 and 2017. They will be regularly followed up at least annually for 5 years to assess all cause deaths, hard clinical events (including cardiovascular death, nonfatal myocardial infarction, nonfatal stroke), and total cardiovascular events (including hard events, unplanned revascularization procedures, unplanned hospitalization for refractory or unstable angina, and for other causes such as stroke, transient ischemic attack, heart failure, or peripheral arterial occlusive disease). The classic and newly defined biosignatures will be compared in patients with and without clinical events during follow-up. The novel biomarkers will be identified via metabolomics analyses. Additionally, psychological personality and lifestyle data will be incorporated to explore the new dimensional views of the complex mechanisms of the disease. Till December 2014, the initial 1663 patients have been successfully enrolled. Among them, 85.93% are male; 36.22% have type 2 diabetes; 64.82% have hypertension; 56.04% are smokers and 20.44% have a family history of CAD. Their lipid profiles are under contemporary medical control with a mean plasma total cholesterol level of 163.51 ± 36.99 mg/dL and a mean low-density lipoprotein cholesterol level of 95.21 ± 29.98 mg/dL.

**Discussion:**

This nationwide study has successfully started to update the contemporary information and to investigate the potential predictors for clinical outcomes of stable CAD patients in Taiwan. The identification of new biomarkers, lifestyle and psychological personality may help to elucidate the complex mechanisms and provide the novel rational to the individual treatment strategies in Asian especially ethnic Chinese patients with CAD.

## Background

Cardiovascular (CV) disease, a common disease in developed countries is associated with increased risk of mortality and morbidity, and is the leading cause of death worldwide. It has beensuggested that atherosclerosis plays a central role in CV disease. A chronic inflammatory process in the vessels leads to vascular damage, atheroma formation, vessel occlusion and even plaque rupture during the progression of atherosclerosis. Despite great efforts including the development of new medical devices, more aggressive revascularization therapies as well as progress in medication, the incidence of events associated with coronary artery disease (CAD) such as acute coronary syndrome (ACS), stroke, cardiac death, and revascularization persists, especially in high-risk CAD patients after coronary intervention. It has been shown that the severity of coronary arterial stenosis could not completely predict the risk of plaque rupture and the occurrence of subsequent thrombosis. A clinical risk score such as the Framingham risk score has been generated to predict cardiac risk, but it cannot reveal the whole disease risk for an Asian population and may lose its predictive value in a population with intermediate risk. The geographic heterogeneity, the genetic susceptibility, dietary habit and environmental risk exposure further increased the complexity of using same risk scoring to predict future risk for patients with CAD. It then becomes important to reassess the clinical profile and the diagnostic and treatment strategies in different cohorts, and to identify indicators for high-risk patients both in the general and the local clinical background.

Disease-specific biosignatures have a biological characteristic that can be measured and evaluated objectively as an indicator of normal biological process, pathogenic process, or pharmacological response to a therapeutic intervention [1]. Biomarkers, as a part of the biosignatures, can be used for a disease at different stages which may be associated with its onset, clinical course, or response to treatment [2–4]. For CV diseases, circulating biomarkers that have been incorporated into clinical practice are mainly used asprognostic markers, and have been shown to have value in addition to classic CV risk factors, which include N-terminal pro-B-type Natriuretic Peptide (NT-proBNP) for heart failure [5], glycated haemoglobin (HbA1c) for glycaemic control in diabetes [6], high-sensitivity TroponinI [7], and high-sensitivity C-Reactive Protein (hs-CRP) for CV risk prediction [8]. Among them, inflammatory related biomarkers such as hs-CRP have been shown useful particularly for CAD patients with intermediate risk [8]. However, further data are still required to examine the specificity and efficacy of these new biomarkers for their clinical implication in different disease severity and in patients of different races in the world.

On the other hand, current treatment of CV disease adheres to tight guidelines. While classic risk factors such as blood pressure, sugar and lipid profile are treated accordingly, future adverse event rate remains high in some well-controlled patients, indicating the possibility of residual risks that need to be targeted, and which may also be related to the lack of more specific and powerful prognostic biomarkers to identify those patients at particular risk. For example, the interaction between personal factors, life styles, and classic risk factors may be complex. On the other hand, the identification of inflammatory biomarkers may be of value to follow the progression of vascular inflammation in atherosclerosis. Therefore, the role of clinical consortia and biospecimen banks is crucial for further investigations. Recent studies have also pointed to the potential of new strategies such as metabolomics or proteomics for novel biomarkers in the investigation of CV disease [9].

Finally, given the potential difference in diet, culture, and physical background between Western and Asian people, either classic or new biomarkers have not been well investigated for clinical outcomes in Asian patients especially ethnic Chinese with stable CAD. Therefore, we conduct a prospective follow-up study on a national basis to evaluate the demographic data, clinical profiles, and treatment strategies for future CV events in stable CAD patients under contemporary treatment in Taiwan. The collected data will be also used to stratify patients at particular future risk by identifying new biosignature profiles such as novel inflammatory biomarkers and the novel biomarkers via metabolomics approach. Here we present the study design/protocol together with the baseline data including demographic characteristics, clinical profiles, medical treatment, and so on in initial 1663 patients enrolled.

## Methods

### Organization

A consortium has been organized to conduct this study. Principle investigator meeting of consortium has been held every two months to discuss any queries about the protocol setting, IRB review process, and cases recruitment. In addition, Professor Ueng-Cheng Yang in National Yang-Ming University and Professor Wen-Harn Pan in Academia Sinica helped to build up the Clinical Informatics & Management System and sample collecting protocol for our consortium. Training courses for data collection were hold for four times. The objectives of the training sessions were not only to produce an instrument for this study but also to instruct the CAD in the principles of instrument design and to elaborate an instrument that could be standardized for use in later CAD research projects. All information will be recorded and blood/urine sample are stored in a central Laboratory in National Yang-Ming University, Taipei, Taiwan.

### Study protocol

#### Patient enrollment

A series of stable CAD patients was initially evaluated in nine different medical centers located in Northern, Central, Southern, and Eastern Taiwan. The patients were initially evaluated if they had a history of significant CAD, as documented by coronary angiogram, a history of myocardial infarction as evidenced by 12-lead electrocardiography (ECG) or hospitalization, or a history of angina with ischemic ECG changes or positive response to stress test. The patients were enrolled only if (1) they had received successful percutaneous coronary intervention (PCI) with either coronary stenting or balloon angioplasty at least once previously, and (2) they had been stable on medical treatment for at least 1 month before enrollment.

#### Exclusion criteria

Patients were excluded if (1) they had been hospitalized for unstable angina, acute coronary syndrome, acute myocardial infarction, acute cerebrovascular events, or other acute cardiovascular events within the 3 months prior to enrollment, (2) they planned to receive further coronary revascularization or interventional procedures for other CV diseases during the following one year period, (3) they had significant malignancy or tumor diseases requiring advanced medical or surgical therapy or both in the following one year, (4) they had other major systemic diseases requiring hospitalization or operation in the following one year, or (5) they were unable or unwilling to be followed up during the following one year period. Additionally, patients with life expectancy of <6 months (e.g., malignant metastatic neoplasm), and those receiving treatment with immunosuppressive agents were also excluded.

#### Clinical follow up for adverse cardiovascular events

According to the study protocol, each patient with initially stable conditions under medical treatment is prospectively and regularly followed up in the individual hospital clinics. After enrollment, follow-up data collection will be taken during outpatient clinic visit, if applicable, and approximately every 3 months for the first year and every 6 months starting from the second year after enrollment. Medication is prescribed for each patient individually at the discretion of the treating physician. During follow-up, the occurrence of adverse CV events will be recorded, including all cause deaths, cardiovascular death, nonfatal myocardial infarction, nonfatal stroke, unplanned revascularization procedures, unplanned hospitalization for refractory or unstable angina, and for other causes including stroke, transient ischemic attack, heart failure, or peripheral arterial occlusive disease. Myocardial infarction is confirmed if ischemic symptoms presented with elevated serum cardiac enzyme levels and/or characteristic ECG changes. Coronary revascularization procedures with either PCI or coronary artery bypass grafting surgery are confirmed by medical record review. Stroke is confirmed if there is a new neurologic deficit lasting for at least 24 h with definite imaging evidence of cerebrovascular accident either by MRI or CT scan. The protocol for CV event follow-up is similar to that has been reported in our previous works [10, 11]. Besides, for the second track of long-term follow-up, the assessment of clinical events will be also conducted by some independent national data banks if the patients agree.

### Baseline data collection

#### Personal and clinical profiles at enrollment

After enrollment, specially trained study nurses and qualified cardiologists collected all data prospectively whenever feasible. Baseline characteristics including risk factors such as history of hypertension, diabetes, smoking habit, as well as medications history were collected. Variable medications and dosage information were collected by chart review and structured questionnaire. Biochemical profiles including blood glucose, lipid profile and kidney function were recorded. In addition, healthy lifestyle factors were noted, including a healthy diet, abstinence from smoking, and regular exercise habit. A healthy diet was identified according to the Recommended Food Score, developed in 2000 by Kant et al. [12] which contains more fruits, vegetables, legumes, nuts, reduced-fat dairy products, whole grains, and fish. Abstinence from smoking applied to those whonever smoked, or had quit smoking for more than 6 months. Regular exercise denotes exercise episodeslasting for at least 30 min, more than five times a week.

#### Personality assessment

In addition to baseline biochemical data and lifestyle information collected, all patients were asked to complete the Type D Scale for Taiwan (DS14-T) after enrollment. This version was translated from DS14 and has been validated for the Taiwanese population [9]. The reliability of Type D assessment in Taiwan is good, with Cronbach’s α for negative affectivity and social inhibition of 0.86 and 0.79 respectively, [13] and adequate construct validity and can be considered equivalent to the Taiwanese version [13]. In short, this instrument is a measure of negative affectivity (e.g., “I often make a fuss about unimportant things” and “I take a gloomy view of things”) and social inhibition (e.g., “I make contact easily when I meet people” and “I find it hard to start a conversation”) with seven five-point Likert-scaled items ranging from 0 = false to 4 = true for each subscale and is regarded as the current standard for identifying the Type D pattern [14]. Patients scoring high on both subscales, according to a standardized cut-off score ≥ 10, are classified as having a Type D personality [15].

#### Blood and urine sampling

After enrollment, 20 mL of blood from peripheral vessels and 10 mL urine were collected. Sampling collection was at the time of enrollment, and every 3 months thereafter in 3-month intervals during outpatient clinic visits. These samples were stored in a −80 °C refrigerator until analysis for biomarkers and metabolomic study. Patients were excluded if they had ingested any drugs with antioxidant activity, vitamins, or food additives within the 4 weeks prior toblood/urine sampling.

### Established and new BiomarkerMeasurement

#### Established biochemical markers for atherosclerotic cardiovascular disease

Biological markers provide quantitative information reflecting biological processes, disease state, or response to treatment in disease management. In the current study, we undertake to investigatea series of established biochemical markers, including but not limited to, the following: (1) high sensitivity C−Reactive Protein (hsCRP); (2) Adiponectin; (3) Matrix Metalloproteinase-3; (4) Matrix Metalloproteinase-9; (5) Interleukin-6; (6) Fibrinogen; (7) Lp-PLA2; (8) Tumor Necrosis Factor-α. Besides these established biochemical markers we attempted to validate another series ofnew potential bio-signature markers for cardiovascular disease.

#### New inflammatory biomarkers for cardiovascular disease

Biochemical marker spotentially associated with the atherosclerosis processwere selected, in particular those related to inflammation including but not limited to the following: (1) Fatty Acid Binding Protein; (2) soluble Vascular Cellular Adhesion Molecule-1; (3) soluble Intercellular Adhesion Molecule-1; (4) Carbohydrate Antigen 125; (5) Monocyte Chemotactic Protein-1; (6) Cardiac Troponin I; (7) CK-MB; (8) NT-proBNP; (9) C-type Natriuretic Peptide; (10) Fetal Heart Binding Protein; (11) Neutrophil Gelatinase-Associated Lipocalin; (12) Chemokine (C-X-C motif) ligand 6; (13) Chemokine (C-X-C motif) ligand 16; (14) Tumor Necrosis Factor ligand superfamily member 14; (15) Oncostatin M; and (16) Myeloperoxidase.

Both the above established and potentially newbiomarkers will be measured sequentially to determine any correlation with the development of future clinical events once the adequate clinical outcomes are identified in the next 5 years.

### Metabolomic study for novel biomarkers

The plasma and urine samples will be frozen in liquid nitrogen immediately and stored at −80 °C to prevent any metabolic decay until further metabolomic analysis. Once more than 50 major cardiovascular events have been identified during follow-up, which may take around 2 years for the initially enrolled 1000 study patients, the metabolomic studies of the plasma and urine will be chronologically performed in the matched CAD cases with major cardiovascular events and those without events during the follow-up period. The biological pathways and clinical biomarkers valuable for predicting the major cardiovascular events of atherosclerotic vascular diseases will be investigated.

Qualitative and quantitative UHPLC -QTOF, GC-MS, LC-QQQ and GCXGC-TOF-MS analysis will be performed at the Metabolomics Core Laboratory. To confirm identified metabolite markers, we will develop a highly sensitive and specific isotope dilution GC-MS or LC-MS method for accurately quantifying the metabolite from biospecimens. Concentration of target markers will be accurately quantified.

### Statistics

A comparison will be drawn between subjects with existing CVD and those who newly develop cardiovascular events during the follow-up period. Quantitative variables will be expressed as mean and standard deviation in the presence of normal or median distribution, and interquartile range in the presence of asymmetric distribution. Qualitative variables will be presented in both absolute frequencies (number of patients) and relative frequencies (percentage). Comparisons of continuous variables between groups will beperformed by ANOVA test, while subgroup comparisons of categorical variables will be assessed by *χ*2 or Fisher’s exact test. The primary, secondary, and tertiary outcomes will be described by an overall percentage, taking all centers into consideration, and the percentage described in each center. These will also be expressed by means of proportions and their confidence intervals of 95%. Where there is great variability in prescription, a weighted average variance at each center will be generated. For regression models, we will report the odds ratio (logistic regression) or hazard ratio (for the regression of Cox proportional hazards), the corresponding standard error, the confidence intervals of 95%, and p values. Additionally, we will report the p values up to three decimals; however, places with p values below 0.001 are reported as p < 0.001. In all the tests, we will use the two-tailed alpha significance level of 0.05.

## Baseline data of initial 1663 patients enrolled

### Baseline patient characteristics

In this study, up to 2500 patients would be enrolled over 5 years according to the schedule. From September 2012 to December 2014, initial 1663 patients with a history of PCI have been successfully enrolled in the first 2.5 years. All patients gave written informed consent before participating in the study. Among them, 1429 (85.93%) were male, 602 (36.22%) had diabetes; 1078 (64.82%) had hypertension; 932 (56.04%) were smokers and 340 (20.44%) had a family history of HCVD. The baseline characteristics of the overall population are provided in Table 1.

**Table 1:** Baseline demographic data of CAD patients enrolled till December 2014

Number of patients	1663
Age at enrollment, yrs	63.49 ± 11.89
Male, n (%)	1429 (85.93)
Body mass index, (kg/m^2^)	26.58 ± 4.4
Waist, cm	93.68 ± 10
Buttocks, cm	99.45 ± 8.57
Waist/buttocks ratio	0.94 ± 0.08
Systolic blood pressure, mmHg	129.82 ± 17.2
Diastolic blood pressure, mmHg	74.85 ± 11.89
Hypertension, n (%)	1078 (64.82)
Diabetes, n (%)	602 (36.22)
Smoking, n (%)	932 (56.04)
Family history of CAD	340 (20.44)
Previous ischemic stroke, n (%)	50 (3.01)
Target lesion in coronary artery (n = 1114)	
Left main artery, n (%)	20 (1.80)
Left anterior descending artery, n (%)	486 (43.63)
Left circumflex artery, n (%)	166 (14.90)
Right coronary artery, n (%)	304 (27.29)
Diameters and length of stents (n = 971)	
Stent Diameters, mm	
Mean ± SD	2.64 ± 0.68
Median (IQR)	3 (2–3)
Stent length, mm	
Mean ± SD	21.99 ± 6.92
Median (IQR)	22 (18–26)
Left ventricular EF, % (n = 1014)	
Mean ± SD	58.67 ± 21.7
Median (IQR)	61 (51–69)

*CAD* coronary artery disease, *EF* ejection fraction (calculated by echocardiography or ventriculography), *mm* minimeter, *n* patient number, *SD* standard deviation

As to the target lesion of coronary artery disease, 43.63% involved the left anterior descending artery, 27.29% the right coronary artery and 14.9% the left circumflex artery. Among the stent information collected, the mean stent diameter was 2.64 ± 0.68 mm with an average length of 21.99 ± 6.92 mm. An echocardiogram was performed in 1014 (61%) of the 1663 CAD patients and their mean left ventricular ejection fraction (58.67 ± 21.7%) was in the normal range.

### Baseline biochemical profiles

Table 2 shows the baseline parameters and biochemical profiles of CAD patients enrolled in this cohort. Total cholesterol was 163.51 ± 36.99 mg/dL and low-density lipoprotein (LDL) cholesterol was 95.21 ± 29.98 mg/dL, showing these patients were treated well and the average LDL value achieved the ATP III/NCEP target of optimal LDL level (<100 mg/dL) [16, 17]. The values of serum high-density lipoprotein (HDL)- cholesterol and triglyceride were 41.63 ± 10.51 mg/dL and 139.76 ± 88.31 mg/dL, respectively.

**Table 2:** Baseline blood biochemical profile at enrollment

Glucose, mg/dL	
Mean ± SD	120.22 ± 42.96
Median (IQR)	107 (95–131)
Cholesterol, mg/dL	
Mean ± SD	163.51 ± 36.99
Median (IQR)	158 (137–185)
LDL-cholesterol, mg/dL	
Mean ± SD	95.21 ± 29.98
Median (IQR)	91 (74–112.2)
HDL-cholesterol, mg/dL	
Mean ± SD	41.63 ± 10.51
Median (IQR)	40 (34–47)
Triglyceride, mg/dL	
Mean ± SD	139.76 ± 88.31
Median (IQR)	116 (84–169)
Serum creatinine, mg/dL	
Mean ± SD	1.3 ± 1.3
Median (IQR)	1.02 (0.86–1.25)
eGFR	
Mean ± SD	76.72 ± 31.05
Median (IQR)	75.66 (60.05–92.63)
White blood cells,/CUMM	
Mean ± SD	7556.08 ± 2345.17
Median (IQR)	7145 (5990–8600)
Hemoglobin, g/dL	
Mean ± SD	13.73 ± 1.83
Median (IQR)	13.9 (12.7–15)
Platelet,/CUMM	
Mean ± SD	215254.96 ± 55152.1
Median (IQR)	211000 (176000–250000)

*eGFR* estimated glomerular fraction rate, *HDL* high density lipoprotein, *LDL* low density lipoprotein

### Baseline medication profiles

Table 3 presents the medication used in thestudy cohort. Anti-platelet agents were prescribed in 93% of patients; anticoagulant in 15.88%; Beta-blocker in 64.86%; diuretics in 13.29%; dihydropyridine (DHP) calcium channel blockers in 32.79%; calcium-blockers (non-DHP) in 37.20%, and RAS blocking agents [angiotensin-converting enzyme (ACE) inhibitors or angiotensin receptor blockers (ARBs) in 62.56%. To our interest, although statin was suggested for all patients with coronary disease, only 73.67% of patients took statin during enrollment. Furthermore, proton pump inhibitors were prescribed in 5.25%. All data will be analyzed for drug interactions in the future.

**Table 3:** Medication treatment at enrollment

Anti-platelet, n (%)	1540 (93)
Anti-coagulant, n (%)	263 (15.88)
ACE-inhibitor, n (%)	356 (21.50)
ARB, n (%)	686 (41.43)
ACE inhibitor/ARB, n (%)	1036 (62.56)
Beta-blockers, n (%)	1074 (64.86)
Calcium-blocker, n (%)	616 (37.20)
Calcium-blocker (DHP), n (%)	543 (32.79)
Diuretics, n (%)	220 (13.29)
Oral nitrates, n (%)	753 (45.47)
Statin, n (%)	1220 (73.67)
PPI, n (%)	87 (5.25)

*ACE* angiotensin-converting enzyme, *ARB* angiotensin receptor blocker, *DHP* dihydropyridine, *PPI* proton pump inhibitor

### Baseline lifestyleand dietary supplementprofiles

To further investigate the effect of lifestyle modification strategies and physiological personality in mitigating future risk, exercise habit and whether diet control followed the ATP III guideline were recorded andare shown in Table 4. Of the study patients, 64.05% were on diet control following the recommendations of ATP III for CAD patients. Exercise frequency has been reported by 886 (51.74%) of enrolled patients, with 54.42% reporting more than 30 min of moderate-intensity exercise 5 days a week. Additional information regarding dietary supplements as well as Chinese medicinal herbs is also shown in Table 4. Dietary supplements were reported by557 (33.53%) study patients, with 446 (79.50%) taking cornmeal, 19.03% taking fish oil, 5.76% Anka, 5.40% Gingko; 5.92% Natto and 5.23% shark cartilage. The incidence of type D personality was 9.63% in the study patients.

**Table 4:** Diet, life style, and personality information of study cohort

Diet control following NECP guidelines for patients with CAD, n (%)	1053 (64.05)
Regular exercise habit, n (%)	860 (51.74)
Exercise frequency	
< 1 times/week, n (%)	63 (7.33)
1–2 times/week, n (%)	123 (14.30)
3–5 times/week, n (%)	198 (23.02)
> 5 times/week, n (%)	468 (54.42)
Use of Chinese medicinal herbs and dietary supplement, n (%)	557 (33.53)
Anka, n (%)	32 (5.76)
Fish oil, n (%)	106 (19.03)
Phytosterols or other stanols, n (%)	3 (0.54)
Cornmeal, n (%)	446 (79.50)
Gingko, n (%)	30 (5.40)
Natto, n (%)	33 (5.92)
Garlic essence, n (%)	11 (1.98)
Grape seed, n (%)	13 (2.35)
Grapefruit juice, n (%)	8 (1.44)
Shark cartilage, n (%)	29 (5.23)
Fur seal oil	6 (1.08)
Type D personality, n (%)	160 (9.63)

*CAD* coronary artery disease

## Discussion

In recent decades, atherosclerosis CV disease has become one of the leading causes of morbidity and mortality in the world. Despite great efforts to reduce risk including medical or primary prevention strategies, the risk remains high and new therapeutic strategies are still needed both in the western and the Asian patients. The current study aims to provide detailed information about long-term outcome in secondary prevention for stable patients with coronary intervention. Besides, it also aims to investigate possible new markers either independently or concomitantly pointing to the occurrence of adverse cardiovascular events. Findings from this study will not only provide new indicators of potential association between secondary prevention and future adverse events but also help to improve our understanding about the effective therapeutic targets as well as strategy to retard or prevent the progression of disease after revascularization.

Among the contemporary pharmacological therapeutic strategy, statins have been clearly shown to be potentially lifesaving medications and are widely used in patients with CV disease. Secondary and primary prevention trials have demonstrated that lipid lowering with statin can reduce morbidity and mortality of CV disease in both western and Asian (mainly Japanese) cohorts [18–20]. Further, a target of serum LDL level <70 mg/dL has been suggested for very high-risk patients according to the findings of some clinical trials mainly in western cohorts [21]. Recently, the 2013 ACC/AHA cholesterol guidelines further identified patients in the four statin benefit groups which included clinical atherosclerotic cardiovascular disease (ASCVD), diabetes, high LDL level (>190 mg/dL) and those with an ASCVD score ≥7.5%, [22] indicating the importance of statin therapy in CV protection. However, most of the above therapeutic strategy and targets are set mainly for western patients. Given the lack of adequate evidence, it is not known if these guidelines could be also feasible to the clinical implication in Asian especially ethnic Chinese cohorts. In this study, while 93% of the study patients received at least one antiplatelet including aspirin and others, there are 73% of the patients on regular statin treatment in this study. The baseline mean plasma LDL level is around 95 mg/dL either with or without statin treatment, which remains higher than the recommended target in patients with established CV disease (≤70 mg/dL). However, it is similar to the LDL values reported in the recently published European registry for secondary prevention study (CICD-PIOT), in which the LDL value is around 91.6 to 110.4 mg/dL, [23] revealing the LDL level for secondary prevention in the real world nowadays. Furthermore, a previous lipid registry for CV disease in Taiwan has reported the achieved mean LDL cholesterol level was 101.49 mg/dL in another secondary prevention CAD cohort during 2010 and 2011 [24]. In fact, the Taiwanese national insurance reimbursement policy have recommended to keep serum total cholesterol level <170 mg/dL and the serum LDL level <100 mg/dL for secondary prevention cohort since 2013. It is clearly demonstrated that the cholesterol control by statin use, as recommended by either global lipid guidelines or national insurance reimbursement policy, has much improved in Taiwan in the last 5 years. Similar findings of the trend of increasing statin use have also been observed in other cohort studies. Park et al. investigated the long-term outcome of left main coronary artery intervention classified by time periods: wave 1 (1995–1998), wave 2 (2003–2006), and wave 3 (2007–2010). The prescription rate of statins has progressively increased from 18.1% (wave 1), to 64.5% (wave 2) and 91.7% (wave3), [25], which confirmed the changing concept of stain use in CAD patients.

Renin-angiotensin system (RAS) is an important mediator of blood volume, arterial pressure, and cardiac and vascular function in CV system. RAS blocking agents (including ACE inhibitors and ARBs) can prevent CV disease by lowering blood pressure and by vascular protection. They have therefore been suggested as the first-line treatment for CV patients with target organ damage. The benefits of RAS blocking agents in the primary and secondary prevention of CAD have been well established [26]. In the current study, while the achieved systolic (130 mmHg) and diastolic blood pressure (75 mmHg) are quite acceptable, the use of renin-angiotensin system (RAS) blocking agents (ACE inhibitors and ARBs) and beta adrenergic blockers are above 60%. Besides, the higher prescription rate of ARBs than that of ACE inhibitors has been also observed in the Biosignature CAD cohort, which is probably due to the better tolerance of ARB use in the Asian population. Interestingly, the increasing use of ACE inhibitor/ARB was also observed in Park’s 25 year-observation, with an increase from 11.9% (wave 1) to 26% (wave 3), supporting the importance of the use of RAS blocking agents in CAD patients [25]. The high prescription rate of ACE inhibitor/ARB in the Biosignature CAD cohort reflects the improvement of evidence-based treatment in CV disease, which is also essential to the improvement of long-term outcome in high-risk CAD patients.

Notably, while the classic risk factors such as lipid profile, blood pressure, and blood sugar have been adequately controlled, the risk of developing a future event may remain high especially in the high-risk CAD patients. Given the potential importance of inflammation in the development and progression of atherosclerosis CV disease, other non-classic risk factors such as lifestyle and environment exposure that might directly or indirectly lead to vascular inflammation/injury should be considered. There is no clear information for the role of lifestyle in secondary prevention for high-risk CAD patients after PCI. Thus, in this study, lifestyle data is collected, which will be investigated to evaluate the potential impact of healthy lifestyle factors including healthy diet, nonsmoking and active exercise on long-term clinical outcomes of the CAD patients. Given the popularity of healthy food not only in western world but also in Asian countries, we also incorporated patient data regarding the use of Chinese medicinal herbs and dietary supplements into the biosignature CAD cohort study. In contrast to the well-known nutrients, these supplements are complex and many potential mechanisms including antioxidant properties, regulation of the inflammatory response, and the proliferation/differentiation of immune cells have been proposed for them. To our interest, there have been few data about the CV benefits of Chinese medical herbs and supplements in CAD patients. In this cohort study, the use of Chinese medical herbs and supplements will be systemically evaluated to provide advanced clues for their potential impact on secondary prevention in CAD patients.

In addition to lifestyle factors, personality trait such as type D personality was included in this study. Personality traits, such as type A (high strung), type B (easy going), and type D (distressed type), have been reported to be variously associated with different CV diseases [27–29]. Recent evidence has pointed to closer connections of personality traits, especially type D personality, with CV diseases. Type D personality represents the effects of two personality traits: negative affectivity (NA, the tendency to experience negative emotions) and social inhibition (SI, the tendency to inhibit the expression of emotions [30]. It has been observed that type D personality might be associated with poor clinical outcomes in patients with congestive heart failure or peripheral arterial occlusive disease, after receiving open heart surgery and after receiving an implantable cardioverter-defibrillator [31]. Accordingly, type D personality may be considered an important risk factor that should be taken into account for risk stratification in some CV diseases. So far, the majority of the international studies on type D personality have indicated that the prevalence of type D personality is between 15 and 29% in CV patients [32, 33]. However, the prevalence of type D personality and its role in long-term outcome in an Asian population remains undetermined. According to the current baseline data, the prevalence of type D personality is around 9 ~ 10% in our study patients. Our study may provide important information on psychological issues in CAD patients.

Finally and most importantly, in this study, serial blood samples will be collected both at baseline and during regular follow-up to evaluate the baseline and the dynamic changes of inflammatory biomarkers for the development of clinical outcomes. The classic and well-established serum biomarkers including hs-CRP and others will be evaluated for both confirmation and comparison. On the other hand, serial new inflammatory biomarkers such as some cytokines, chemokines and inflammatory ligands that are less related to clinical atherosclerosis disease will be evaluated to identify their potential prognostic role for clinical outcomes in our CAD patients. The new biomarkers that could improve the prognostic value of well-established biomarkers such as hs-CRP will be further investigated. Once the clinical significance could be confirmed by replication, further new projects will be initiated with both in vitro and in vivo experiments to identify the potential mechanisms and new therapeutic targets. Besides, to further define the novel biosignatures as well as novel mechanisms, another line of metablomic study will be also done in selected cases in this study. Taken together, our study focused on secondary prevention to identify the new predictors and to investigate the potential mechanisms and novel therapeutic target in stable patients after PCI even when they are already under contemporary treatment.

### Study limitations

Although the criteria for patient enrollment and the protocol for clinical follow-up have been clearly defined and all cases are recruited by well-train staffs in teaching hospitals, selection bias arising from clinical profile, investigator participation and treatment adherence by patients could not be completely excluded. Furthermore, according to the population distribution, the patients are extensively recruited throughout the whole country in order to represent the people living in Taiwan. However, this is a hospital-based rather than a community-based study. Further investigations may be required to elucidate and adjust the potential geographic variations in therapeutic principle, environmental exposure, patient behavior, and so on. Besides, given the long-term follow-up, a substantial portion of patients may be lost to follow up. Thus, the clinical outcomes of the patients will be also periodically assessed through some national data banks such as the national insurance policy data bank in Taiwan if indicated.

## Conclusions

The biosignature CAD study is a nationwide prospective cohort study aiming to investigate new biosignatures to identify the CAD patients at very high risk in an Asian cohort. Given the comprehensive network in Taiwan, all the patients could be enrolled and closely followed up for 5 years to assess impacts of the classic and non-classic risk factors as well as the standard and novel biomarkers on future clinical events. As the study is ongoing, the baseline data revealed that the patients are in stable condition by standard contemporary treatment at enrollment. The investigation on new biosignatures including inflammatory markers, personality traits and environment factors will further improve our understandings of the complex atherosclerosis disease and advance the therapeutic strategies of stable CAD in Asian patients especially the ethnic Chinese in Taiwan.

## Abbreviations

ACE: Angiotensin-converting enzyme
ARB: Angiotensin receptor blocker
BP: Blood pressure
CAD: Coronary artery disease
DHP: Dihydropyridine
EF: Ejection fraction
eGFR: Estimated glomerular filtration rate
HDL: High density lipoprotein
hsCRP: High sensitivity C-Reactive Protein
LDL: Low density lipoprotein
PPI: Proton pump inhibitor
RAS: Renin–angiotensin system
